# Adhesive curing through low-voltage activation

**DOI:** 10.1038/ncomms9050

**Published:** 2015-08-18

**Authors:** Jianfeng Ping, Feng Gao, Jian Lin Chen, Richard D. Webster, Terry W. J. Steele

**Affiliations:** 1 School of Materials Science and Engineering, College of Engineering, Nanyang Technological University, 50 Nanyang Avenue, Singapore 639798, Singapore; 2 School of Biosystems Engineering and Food Science, Zhejiang University, 866 Yuhangtang Road, Hangzhou 310058, China; 3 Division of Chemistry and Biological Chemistry, School of Physical and Mathematical Sciences, Nanyang Technological University, Singapore 637371, Singapore

## Abstract

Instant curing adhesives typically fall within three categories, being activated by either light (photocuring), heat (thermocuring) or chemical means. These curing strategies limit applications to specific substrates and can only be activated under certain conditions. Here we present the development of an instant curing adhesive through low-voltage activation. The electrocuring adhesive is synthesized by grafting carbene precursors on polyamidoamine dendrimers and dissolving in aqueous solvents to form viscous gels. The electrocuring adhesives are activated at −2 V versus Ag/AgCl, allowing tunable crosslinking within the dendrimer matrix and on both electrode surfaces. As the applied voltage discontinued, crosslinking immediately terminated. Thus, crosslinking initiation and propagation are observed to be voltage and time dependent, enabling tuning of both material properties and adhesive strength. The electrocuring adhesive has immediate implications in manufacturing and development of implantable bioadhesives.

## Abstract (web summary)

Existing methods of adhesive curing suffer from restrictions in the circumstances in which they can be employed. Here, the authors design a new method whereby application of a potential difference induces the curing process, increasing the scope for implementation to a whole new range of substrates and conditions.

## Graphical abstract

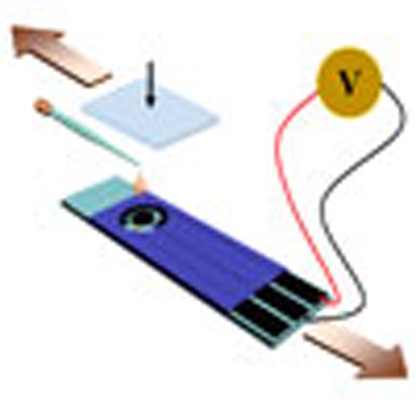

Chemical-curing adhesives (CCA) have become ubiquitous as a joining technique in manufacturing of medical devices, in automotive industries, and in consumer goods. The main advantages of CCA is that they have been formulated to combine inherent adhesive advantages (weight saving and stress distribution) with the advantages of instant fixation (adhesion on demand). Instant fixation is of prime importance, especially if one seeks to replace laborious mechanical fixation techniques such as rivets, screws, and bolts. Despite the importance of instant fixation, little innovation in terms of stimuli activation has been accomplished in the past few decades and most current CCA technologies are activated by either temperature, light, or two-part thermosets.

Snap-cure adhesives are essentially two-part thermoset adhesives with a catalyst additive, that allow curing at a relatively low-temperature activation of 110–125 °C—but they cannot be used for heat-sensitive materials and the catalyst can leach from the adhesive^1^. Polymerized by light-based mechanisms, photoadhesives are typically ultraviolet-cured and require high amounts of photoinitiators, which contribute to manufacturing problems of high dermal sensitivity. They are limited to visible surfaces and transparent substrates, such as coatings and laminates^2^. A ubiquitous ‘instant-cure' adhesive is methyl/ethyl-cyanoacrylate, also known as ‘Superglue'. It has the unique distinction of either forming very strong substrate bonds or not bonding at all. Cyanoacrylates initiate adhesive polymerization via anionic groups on the substrate surface—typically moisture (hydroxyl ions) on most substrates. Their use is limited in manufacturing. Handling is difficult (moisture is everywhere) and the bonding process cannot be end-user activated. Other limitations include their inability to bond rough/acidic surfaces (metals), brittle material properties and low-temperature durability (cured bonds must be kept <70 °C)^3^.

Electrochemically mediated adhesive curing (‘electrocuring') is a concept by which adhesives can be activated on-demand when an applied voltage potential is raised above a certain threshold. As the voltage surpasses this threshold, electrochemically activated functional groups are switched ‘on' and able to crosslink the polymer backbone and substrates that they are to be adhered to. Ideally, this crosslinking would be arrested once the voltage falls below a threshold, allowing the end-user to modify both the bonding strength and adhesive material properties. This type of control would surpass current chemical-curing adhesives.

We are aware of no commercial adhesives or scientific literature that report on-demand adhesives cured by applied voltage-mediated initiation. Electrocuring adhesives would solve many of the limitations described above. They could be utilized on heat-sensitive materials, contain no sensitizing initiators, and their potential versatility may be perfect for in-line manufacturing. For example, they are ideal for automation adhesive applications (extrusion on a molded part, thin-film tapes and so on), as electrocuring is separated in two distinct phases without the maintenance-intensive hardware associated with thermo-/photocuring, or two-part adhesives.

Here we demonstrate curing an adhesive through low-voltage activation or ‘electrocuring' which gives unique end-user properties. Material properties and adhesion strength can be tailored at will. The flexible electrocuring adhesive is possible because of electrochemically activated carbene precursor functional groups capped on a PAMAM dendrimer. Low-voltage activation triggers the carbene groups to crosslink electrode surfaces and neighbouring dendrimers.

## Results

### Selection of an electrocuring carbene precursor

**Figure 1:**
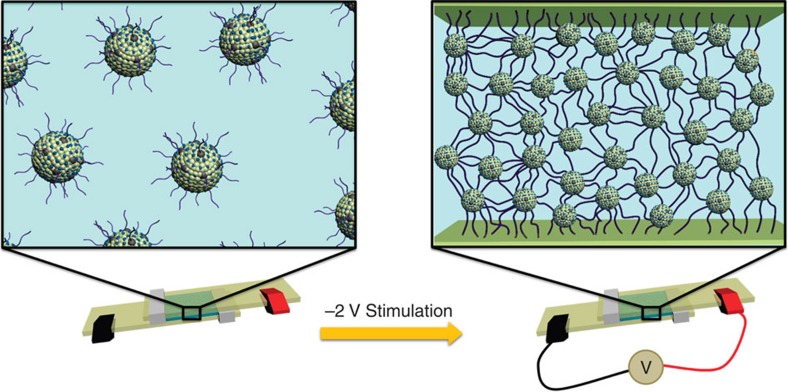
Concept of electrocuring adhesive. Low-voltage activation allows polymer and substrate crosslinking.

The ability to crosslink indiscriminately is a characteristic of free-radical chemistry. There are a host of free-radical precursors that can be triggered via reductive electrochemistry. Diazonium salts and vinylics may be the most well-known, but vinylics have poor surface crosslinking and only the initiation step is controlled via electrochemistry^4^. Diazonium salts are highly versatile in the compounds they can crosslink—which spans from inorganic silicon, dielectric polymers of Teflon and polyethylene terephthalate to precious metals^5^,^6^,^7^. However, high pH instability and acidic working conditions limit their usage to pH<3 (refs 8, 9). Carbene precursors are similarly versatile as diazonium groups in terms of crosslinking, and are known to have high crosslinking efficiencies and relatively fast reaction times^10^,^11^. However, only a few reports exist of carbene precursor activation by electrochemical methods^12^,^13^,^14^,^15^. Due to its ease in polymer grafting, commercial availability, and previous known electrochemical activation^13^, the carbene precursor 3-[4-(bromomethyl)phenyl]-3-(trifluoromethyl)-diazirine (denoted as aryl-diazirine) was selected as a promising electrocuring functional group, capable of crosslinking on demand (see Fig. 1).

### Aryl-diazirine is electrochemically reversibly reduced

**Figure 2:**
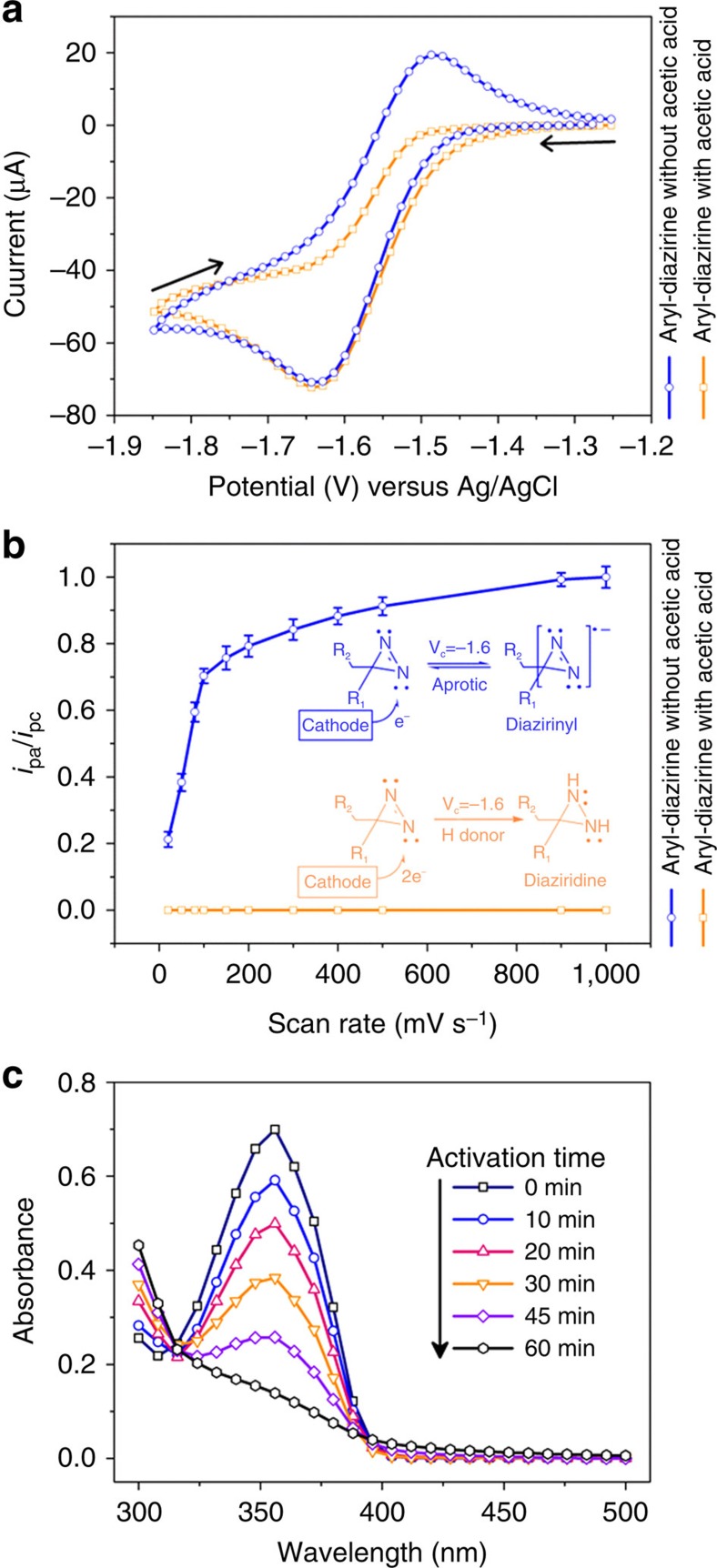
Electrochemical behaviour of aryl-diazirine. (**a**) Cyclic voltammograms recorded for 1 mM aryl-diazirine in the absence and presence of 1.0% acetic acid (proton donor) in acetonitrile containing 0.1 M tetraethylammonium perchlorate at a glassy carbon electrode (d. 3 mm). Scan rate: 100 mV s^−1^. (**b**) *i*_pa_/*i*_pc_ values over a range of scan rates. The *i*_pa_ represents anodic peak current and *i*_pc_ represents cathodic peak current. (**c**) Absorbance spectra of 1 mM aryl-diazirine in acetonitrile containing 0.1 M tetraethylammonium perchlorate on various electrostimulation times with an applied voltage of −2.0 V versus Ag/AgCl at a glassy carbon electrode (diameter, 10 mm).

Electrochemical characterization of aryl-diazirine in acetonitrile was performed using cyclic voltammetry to determine its reduction potential and degree of chemical reversibility. At a scan rate of 100 mV s^−1^, semi-chemically reversible behaviour is observed (Fig. 2a). The ratio of the oxidative peak current (*i*_pa_) to that of the reduction peak current (*i*_pc_) is <1, indicating that the process is not completely chemically reversible at low scan rates and suggesting that the species formed by initial electron transfers reacts further before it is converted back to the starting material on the reverse scan. Such voltammetric behaviour is similar to that observed for 3-*n*-butylphenyldiazirine^14^, in which the heterogeneous electron transfer step is followed by a homogeneous chemical step. As the scan rate increases up to 1 V s^−1^, the *i*_pa_/*i*_pc_ ratio approaches unity, indicating that the chemical step following initial electron transfer can be outrun, so that the starting material can be completely regenerated from its reduced form at sufficiently fast scan rates. Nevertheless, the cathodic (*E*_p_^red^) to anodic (*E*_p_^ox^) peak-to-peak separation of ∼150 mV at 0.1 V s^−1^ was considerably greater than the 57 mV expected for a one-electron electrochemically reversible process^16^, suggesting relatively slow heterogeneous electron transfer (quasi-reversible behaviour). The reduced species, formed by initial one-electron reduction, is the anion radical of the aryl-diazirine, aryl-diazirinyl, which has previously been investigated with electron-spin resonance spectroscopy^13^. On addition of acetic acid, the voltammogram displayed a chemically irreversible reduction process at all scan rates tested, as shown in Fig. 2a,b, which may be a reduction of the diazirine to diaziridine^15^. Mass spectrometry positive-ion analysis of the electrochemically treated aryl-diazirine confirmed the presence of aryl-diaziridine at m/z ratios of 280 and 282 (bromine, present in aryl-diaziridine, naturally exists as two isotopes ^79^Br and ^81^Br). Ultraviolet spectroscopy was used to monitor the electrolysis of aryl-diazirine at a glassy carbon electrode. Fig. 2c displays the ultraviolet absorbance and aryl-diazirine's subsequent absorbance decrease with upon electrolysis. The solution was visually observed to turn from a clear transparent solution to a yellow tint during electrolysis.

### Structural design and synthesis of electrocuring adhesive

**Figure 3:**
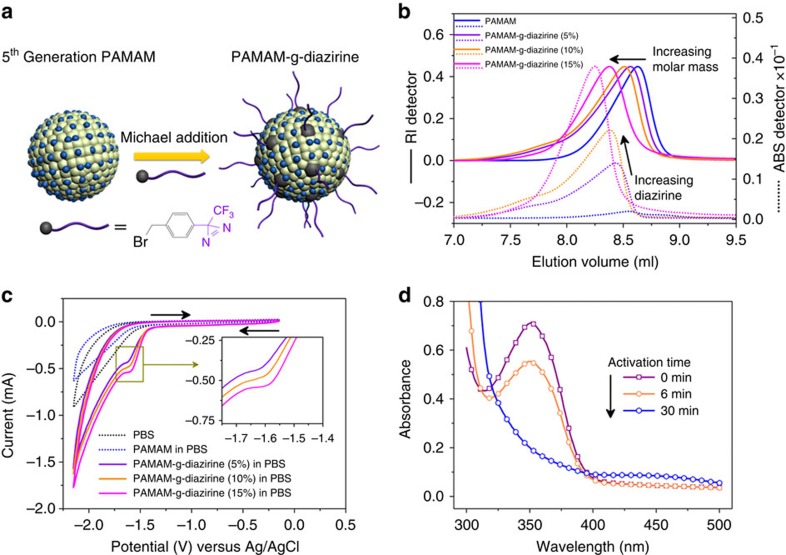
Characterization of PAMAM-g-diazirine conjugates. (**a**) Schematic illustration of the synthesis route of PAMAM-g-diazirine conjugate. (**b**) SEC–MALLS–UV characterization of the fifth generation PAMAM (control) and the subsequently modified PAMAM-g-diazirine conjugates. (**c**) Electrochemical behaviour of PAMAM (control) and PAMAM-g-diazirine conjugates in PBS at a glassy carbon electrode (diameter, 3 mm). Scan rate: 100 mV s^−1^. (**d**) Absorbance spectra of PAMAM-g-diazirine in PBS on different electroactivation times (0, 6 and 30 min) with an applied voltage of −2.0 V at a glassy carbon electrode (diameter, 10 mm).

Chemical adhesion results from the active crosslinking of reactive functional groups to adjacent polymer chains, substrate surfaces or combinations thereof. Linear polymers are more likely to undergo intramolecular (versus intermolecular) crosslinking than branched polymers or dendrimers^17^. Thus, we chose the fifth generation polyamidoamine (PAMAM) dendrimer as it allows a high probability of intermolecular crosslinking by grafting a carbene precursor on to the primary amines present on the macromolecule surface, as seen in Fig. 3a. The aryl-diazirine is grafted onto the PAMAM dendrimer (denoted as PAMAM-g-diazirine) via bromine-primary amine Michael addition. The PAMAM-g-diazirine is characterized through size exclusion chromatography with online multiangle laser light-scattering and ultraviolet detectors (SEC–MALLS–UV). Figure 3b displays a representative SEC–MALLS–UV plot of three PAMAM-g-diazirine conjugates that differ by the ratio of diazirine grafting. Conjugation degree and molar mass data are summarized in Supplementary Table 1. Solid lines (refractive index detector) in Fig. 3b are seen to shift left (increasing weight-averaged molar mass, *M*_w_) as the diazirine conjugation is increased. The dashed lines (350 nm absorbance) overlaying the refractive index detector signals indicate successful grafting of aryl-diazirine and formation of the PAMAM-g-diazirine conjugate.

### Electrochemistry of PAMAM-g-diazirine

Electrochemical reduction of diazirine in alkaline aqueous conditions has been shown to occur in a chemically irreversible process to form the diaziridine^15^. Diazirines grafted on the polymer conjugates are expected to have similar behaviour. Cyclic voltammograms of PAMAM-g-diazirine in PBS (pH 7.4, 0.1 M) are shown in Fig. 3c. A small cathodic peak for the conjugate is found at −1.6 V. Compared with the controls of PBS and PAMAM, the conjugate cathodic peak could be ascribed to the reduction of grafted diazirine. The current of the cathodic peak escalated with conjugation degree of PAMAM-g-diazirine, higher density of current carrier support by phenyl functional groups, or combinations thereof. All three voltammograms of PAMAM-g-diazirine exhibited no oxidation peaks when the scan direction was reversed after first reducing the diazirine at scan rates up to 1 V s^−1^, suggesting no equilibrium of diazirinyl anion radicals. Figure 3d shows the ultraviolet absorption spectrum of PAMAM-g-diazirine under electrolysis conditions. Similar to the electrochemistry of aryl-diazirine (Fig. 2c), the ultraviolet absorbance spectrum of PAMAM-g-diazirine decreases over time as seen in Fig. 3d. However, it should be noted that this extended diazirine reaction is concentration dependent, where electrochemical analysis requires dilute solutions and likely does not reflect the highly concentrated formulations required for shear adhesion measurements.

### Tunable material properties of PAMAM-g-diazirine

**Figure 4:**
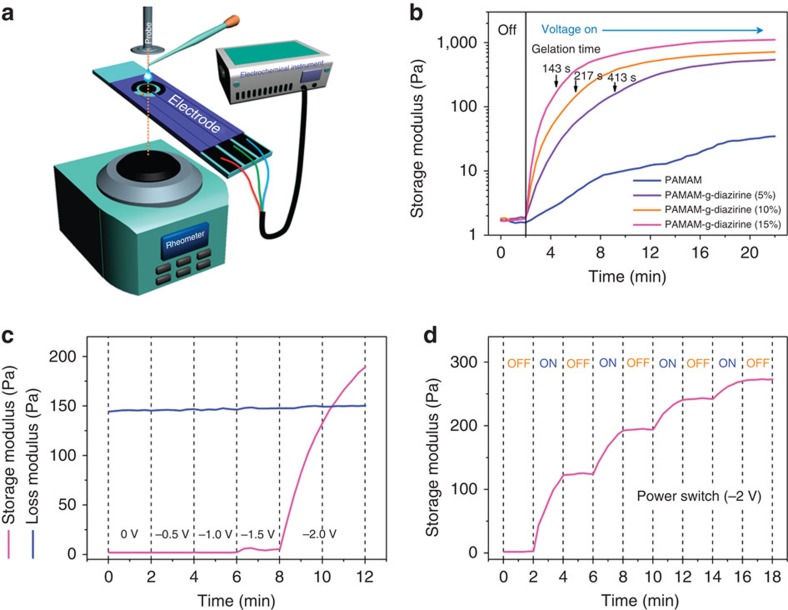
Electrorheological properties of PAMAM-g-diazirine solutions. (**a**) Scheme of real-time oscillatory dynamic rheometry of electro-activated adhesives. (**b**) Storage modulus (G′) with respect to time before and after an applied voltage of −2.0 V versus Ag/AgCl on the disposable Zensor chip. PAMAM-g-diazirine solutions (25 wt% in PBS in all figures) with different conjugation degrees (5, 10 and 15%) were electrocured for 20 min. (**c**) Kinetics of storage modulus (G′) and loss modulus (G″) with respect to magnitude of applied potential versus Ag/AgCl. Gelation point is defined where G′=G″. (**d**) Kinetics of storage modulus with respect to temporal activation of a −2.0 V applied to PAMAM-g-diazirine (15%).

The rheology performance of PAMAM-g-diazirine under −2 V applied potential was investigated with respect to electrochemical activation. This allowed the characterization of the hydrogel mechanical properties in real time via oscillatory dynamic rheometry through parallel-plate geometry. The experimental set-up is demonstrated in Fig. 4a, where PAMAM-g-diazirine rheological analysis is followed through the electrochemical activation (electrocuring) on a disposable Zensor chip with three screen printed electrodes. The storage modulus (G′) and loss modulus (G″) in amplitude tests (Supplementary Fig. 1) were almost stable when the deformation (strain) is 1%. Thus the oscillatory rheometry (1 Hz, 1%) of electrochemically activated PAMAM-g-diazirine conjugate is performed in linear-viscoelastic range. The PAMAM-g-diazirine storage modulus with various diazirine conjugation degrees was measured under ‘OFF' (control) and ‘ON' −2 V activation, as seen in Fig. 4b. Under no voltage activation (‘OFF' region in Fig. 4b), the G′ or storage modulus is stable, hence no crosslinking is initiated or observed (see first 2 min). On an applied voltage, an immediate increase in storage modulus is observed, that displays a logarithmic growth function—a large increase in G′/time (slope) at the onset that quickly decays but never reaches zero. PAMAM-g-diazirine conjugates with higher amounts of grafted diazirine undergo faster changes in G′/time and obtain higher storage modulus with respect to a given amount of time. PAMAM-g-diazirine at 5%, 10% and 15% conjugation in 25% w/v PBS displays a storage modulus of 0.5, 0.7 and 1 kPa after 20-min activation, respectively. In comparison, the storage modulus is comparable to gelatin or liver tissue^18^,^19^. Note that these storage moduli are relatively small only because of the small deformations (1% amplitude) that are applied to the anisotropic hydrogel, as explained in the next section. Gelation time, the point where storage modulus and the loss modulus have equal values (as seen in Fig. 4b), decreases with the increase of grafted diazirine on PAMAM. Using Ohm's law, the resistance of the hydrogel was measured to be 76, 6.9 and 2.9 kΩ for the 5%, 10% and 15% conjugated PAMAM-g-diazirine, respectively, suggesting a percolation threshold is crossed from 5 to 10%. To exclude competing side reactions to the PAMAM-g-diazirine crosslinking mechanism, PAMAM-g-diazirine (15%) was activated in −0.5 V increments, up to −2 V, as displayed in Fig. 4c. No change in properties was seen under −1 V. At −1.5 V, where water electrolysis is applicable, modulus values are slightly shifted, but no crosslinking or increase in modulus is observed. This signal change in modulus within this region is likely due to changes in surface chemistry on the three electrodes. When the voltage exceeds the −1.6 V required (Fig. 3c, inset) for diazirine activation, instantaneous crosslinking is observed as measured by the sharp increase in storage modulus. A simple cyclic power switch is performed to assess the mechanical properties before and after electrochemical activation. At 2-min intervals, −2 V is switched ‘ON' and ‘OFF' for four cycles with the results displayed in Fig. 4d. After the first cycle, the storage modulus is regarded as stable and the crosslinking deemed irreversible (under these limited conditions). This suggests chemical crosslinking by electrochemical activation of diazirine and the change in storage modulus was not subject to other events, for example, PAMAM polarization by electric fields, resistive heating. Note that both crosslinking initiation and propagation were controlled by the electrochemical activation—crosslinking halted under the ‘OFF' conditions. Most on-demand (that is, photocuring) adhesives only control initiation, while propagation continues uncontrollably. The combined data demonstrate that the adhesive mechanical properties (G′, G″ and gelation point) may be tuned by varying stimulation time and diazirine conjugation degree.

### PAMAM-g-diazirine allows tunable shear adhesion strength

**Figure 5 f5:**
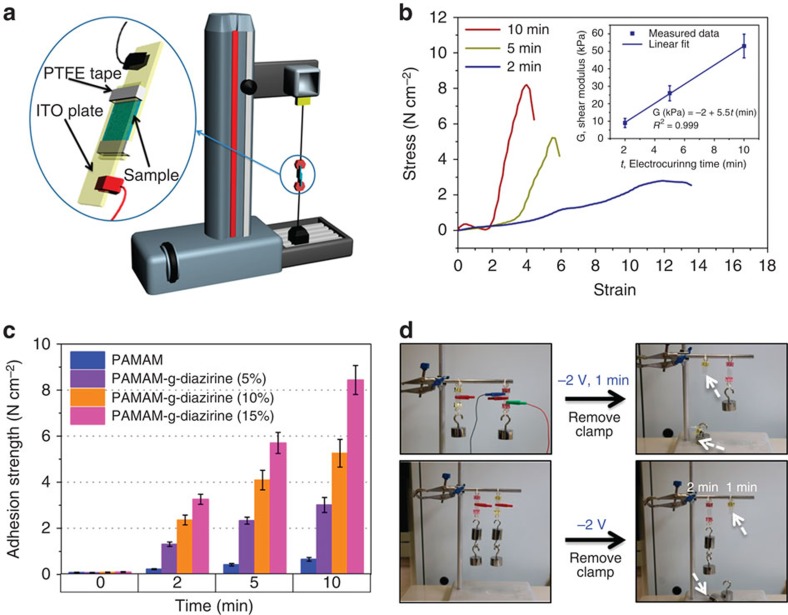
Shear adhesion of electro-activated PAMAM-g-diazirine conjugates. (**a**) Experimental set-up of shear adhesion failure analysis. (**b**) Typical shear stress versus shear strain curves of electrocured PAMAM-g-diazirine (15%) conjugates in PBS with the concentration of 25 wt%. Electrocuring adhesive is sandwiched between two transparent ITO plates with various times of electroactivation at −2 V. Inset: maximum shear modulus (G) was plot against electrocuring time (*t*) with a high linear correlation. (**c**) Comparison of maximum shear stress values obtained across all tested PAMAM-g-diazirine conjugates and electroactivation times. (**d**) Demonstration 1 (top): tunable adhesive properties employing 100-g mass standards (0.98 N force) at 0- and 1-min electrocuring times. With no electroactivation, the inactivated adhesive fails to support the load. Demonstration 2 (bottom): breakaway tuning of electrocuring adhesive properties employing 200-g mass standards (1.96 N force). The electrocuring adhesive time for one formulations of PAMAM-g-diazirine was chosen to predict shear adhesion strength that would allow support of the stress or adhesion failure.

Shear adhesion strength is assessed by sandwiching the PAMAM-g-diazirine hydrogel in between two conductive indium tin oxide (ITO) glass plates, as seen in Fig. 5a. The thickness of the gel is controlled through dielectric PTFE tape, which is *ca*. 90-μm thick. The electrocuring adhesive is stimulated by applying −2 V across the two ITO plates from 0 to 10 min. Shear stress versus shear strain is recorded. Fig. 5b displays a typical result from PAMAM-g-diazirine (15%) with non-linear shear moduli (as measured by the slope). The uncured, pure viscous PAMAM-g-diazirine (15%) shows a flowing, viscoelastic gel at 2-min electrocuring, where G″ and G′ are predicted to be *ca*. equal in magnitude (as seen in Fig. 4b,c). For this time, a maximum shear modulus (G, also known as modulus of rigidity) of 4.8 kPa (at 12 shear strain) is determined. Electrocuring of 5 or 10 min created an elastic-like solid that had maximum shear moduli of 25 kPa (at 5.4 shear strain) and 53 kPa (at 4 shear strain), respectively. Figure 5b (inset) displays as strong linear correlation of shear modulus and electrocuring time, with a linear fit of *R*^2^=0.999. Maximum shear stress attained (adhesion strength) is plotted and summarized for all formulations in Fig. 5c. Adhesion strength and diazirine conjugation also display strong linear correlation with *R*^2^>0.99 for all diazirine conjugates, allowing straightforward prediction of adhesion strength at specific electrocuring timepoints. Thus, adhesion strength could be tuned through *ca*. 1–8 N cm^−2^. To demonstrate the application of the shear adhesion correlations, electrocuring time (1 or 2 min) can be employed to either hold or allow an adhesion failure for specific masses—for example, to design breakaway components required in certain engineering applications. Figure 5d shows that the shear adhesive bonding strength for one formulation could be tuned to allow successful bonding of a stress at 2-min electrocuring or for failure of the same stress at 1-min electrocuring. Cohesive failure was noted for all PAMAM-g-diazirine formulations described above. The shear adhesion between Zensor chips against non-conductive organic (polyester terephthalate, Supplementary Fig. 2a) and inorganic (borosilicate glass, Supplementary Fig. 2b) coverslips was also performed as an initial proof of concept bonding of non-conductive substrates. The Zensor TE100 chips consist of non-metallic carbon electrodes printed on a plastic substrate. PAMAM-g-diazirine (15% conjugation at 25 wt%) was applied between the Zensor chip and the non-conductive substrates. After electrocuring for 10 min, a maximum shear strength of 2–2.5 N cm^−2^ was recorded with the cohesive failure. The hydrogel was observed to cure across the 0.7 cm^2^ application area, despite the limited Zensor TE100 working electrode area of 0.071 sq. cm^20^.

## Discussion

Contrary to popular belief, more durable adhesion results from soft viscoelastic adhesives than hard, highly crosslinked structural adhesives. Adhesives with low moduli and viscoelastic properties—capable of plastic deformation—display superior long-term durability compared with rigid adhesives that are sensitive to shock (that is, pressure sensitive adhesive ‘duct tapes' versus cyanoacrylate ‘superglues'). However, no conventional adhesives allow a stable choice of adhesive modulus and viscoelastic properties towards the substrate at hand. Herein we have demonstrated an electrocuring adhesive that incorporates this flexible tuning of material properites and a novel on-demand method of adhesive activation. The adhesive is designed as a low-molecular weight, flowable low-viscosity liquid that transforms into a crosslinked, polymerized matrix with high molecular mass and high shear-loading potential. The low-viscosity liquid allows good wetting of the substrates while electrocuring is initiated only after adherents are joined and properly positioned.

**Figure 6:**
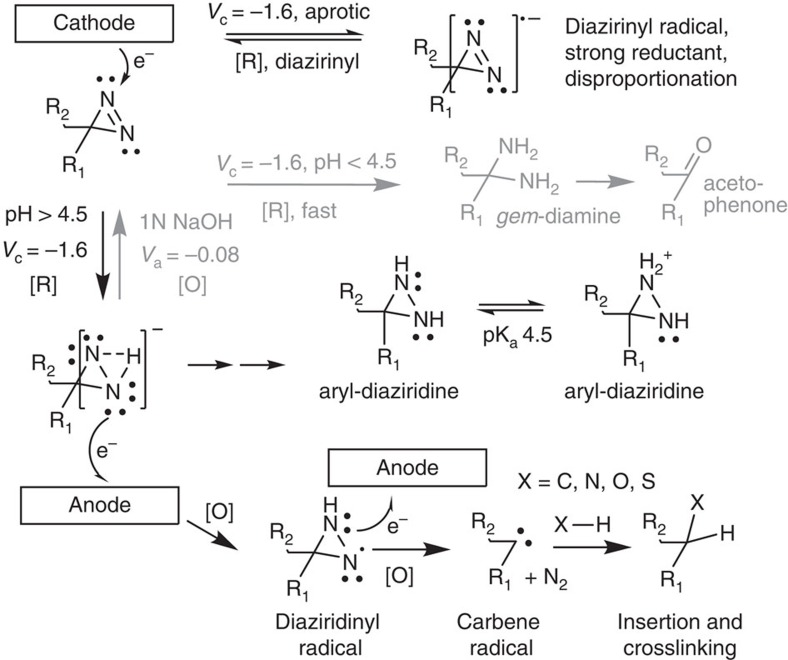
Diazirine electrochemistry and aryl-carbene formation. Depending on the environment, several products are possible for the electrochemical reduction of aryl-substituted diazirine. In aprotic environments, the semistable diazirinyl radical is formed that can act as a strong reducing agents towards other molecules or itself (disproportionation)^12^,^13^,^14^. In acidic protic mediums, diaziridine can be protonated, explaining how the diazirine is reduced into the gem-diamine which under goes hydrolysis to the ketone^15^,^40^. Alternatively, aryl-diaziridine can be oxidized to the original diazirine in caustic pH>11^15^. The aryl-diazirine or one of its negatively charged intermediates (see square scheme in Supplementary Fig. 3) may be oxidized by the positively charged anode into the short lived carbene radical, which inserts itself into X–H species. Any number of elements could be ‘X' including Carbon, Nitrogen, Oxygen or Sulfur.

**Figure 7:**
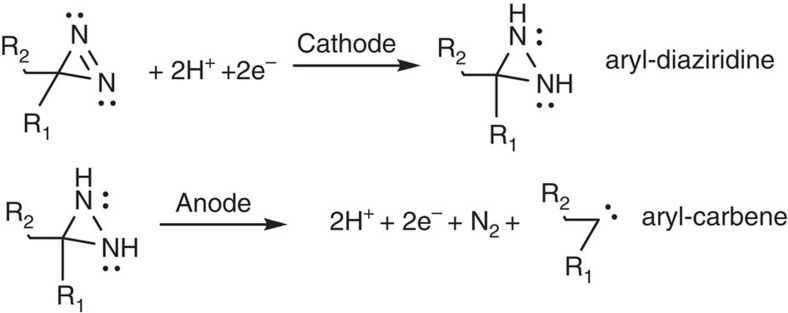
Proposed half-reactions at the cathode and anode. Aryl-diazirine is reduced to aryl-diaziridine with two equivalents of electrons and protons. Aryl-diaziridine is then oxidized to aryl-carbene yielding two equivalents of electrons and protons and one equivalent of diatomic nitrogen.

The electrochemistry of diazirine, diaziridine, and R1R2N2 functional groups has been previously explored in relation to reduction and oxidation under various experimental conditions^12^,^13^,^14^,^15^. A simplified scheme is presented in Fig. 6 that combines previous observations of relevant diazirine/diaziridine chemistry with our proposed mechanism of aryl-diazirine mediated electrocuring. We have avoided acidic environments where diazirine is completely reduced to gem-diamine and acetophenone, although this would allow electrochemically activated crosslinking via Schiff-base chemistry and may allow an improvement over two-part mediated Schiff-base bioadhesives^21^. Under aprotic conditions, single-electron reduction yields the stable and reversible diazirnyl radical (*t*_1/2_≈46 s)^13^, which is itself a strong reductant and may act to propagate reduction within the bulk medium. In the presence of a proton donor, diazirine is quickly and irreversibly reduced to diaziridine^15^. The presence of aryl-diaziridine was confirmed by mass spectrometry and was not found in unreacted aryl-diazirine. Previous investigations have shown that diaziridines are capable of forming diazirinyl radicals with subsequent carbene formation through a light-activated radical oxidizer^22^,^23^,^24^. Others have postulated an aqueous diazirdine-to-carbene mechanism that allows irreversible enzyme inactivation^25^. Thus, we propose that the adhesive properties observed (as shown in Figs 5 and 6) is due to carbene-mediated crosslinking through diaziridine activation by an electrochemically generated radical oxidizer. The exact nature of the radical oxidizer within the PBS can only be speculated, but several pathways exist: (1) direct formation of diaziridinyl radicals via the anode; (2) aminyl radical formation via amine oxidation at the anode^26^,^27^ (primary amines are present on PAMAM); or (3) Radical oxide species generated in PBS by water electrolysis^28^. If radical oxide species are generated, then potential aging problems may arise at the anode or substrate interfaces and may need to be mediated if long-term durability of the joints is required. The proposed half-reactions at the cathode and anode are given in Fig. 7 and are under continued investigation. However, other electroreductive mechanisms of crosslinking are conceivable and cannot be ruled out at this time. Electrochemical reduction of aryl-diazo compounds (the diazo group is a chemically related isomer of diazirine) have yielded crosslinked aryl-azine compounds or carbene anion radicals^12^,^29^.

Electron hopping across PAMAM-g-diazirine/diaziridine allows electrode and intermolecular crosslinking. The formation of carbene radicals allows non-specific intermolecular and substrate crosslinking, which is ideal for an adhesive. Considering the initial hydrogel viscosities (50–150 Pa s or the consistency of ketchup), molar masses of 30 kDa or more, and the propensity to crosslink, it is unlikely that the PAMAM-g-diazirine conjugates are able to instantaneously diffuse from the cathode to the anode surface, which was always *ca*. 100-μm apart. Put another way, the conjugates are fundamentally immobile within the environments and time scales considered herein. However, the available evidence suggests both the cathode and anode each play an important activation step in the proposed carbene mechanism. This presents a paradox: if both the cathode (diazirine → diazirdine) and the anode (diaziridine → carbene) are required, then how does the immobile PAMAM-g-diazirine contact both electrode surfaces? Homogenous electron exchanges (also known as, electron hopping) may explain the propagation of reduction and subsequent diaziridine oxidation across the grafted aryl functional groups and the mostly immobilized PAMAM dendrimer. Indeed, higher densities of aryl functional groups increased current and crosslinking kinetics as seen in Fig. 3c and Fig. 4b, respectively. This process is exemplified in the square scheme and current/resistance plots in Supplementary Fig. 3 and Supplementary Fig. 4, respectively. Alternatively, tunneling is possible through the many peptide branches of the PAMAM dendrimer. This would allow further conduction of electrons via the intramolecular peptide branches. Sensors based on PAMAM dendrimers have been shown to be capable of electron transport^30^,^31^. Thus, electron hopping, tunneling or combinations thereof contributes to the observed bonding to the anode, cathode and bulk crosslinking.

Let us now consider the differences between electrocuring and electropolymerization. The classical electropolymerization (that is, electrochemically induced polymerization) involves one primary electron transfer reaction occurring at the working electrode; this can involve a material being deposited on the electrode surface (such as for a conducting polymer) or it can involve the electrogeneration of a species (such as a metal in a particular oxidation state) that initiates a polymerization reaction of another material^32^. In these situations the polymerization reaction occurs or is initiated at only the working electrode, while the counter electrode (or auxiliary electrode in a three-electrode system) simply acts as the collector or the donor of electrons to balance the overall charge and enable current to flow in the circuit^33^. However, in our electrocuring system, the free radicals (diazirinyl radical generated at cathode and carbene radical generated at anode) generated during electrochemical activation are the functional groups necessary for polymer crosslinking and surface binding. As a result, the polymerization reaction occurs not only at both the surfaces of the cathode and anode, but also in the bulk medium. The rheology and adhesive data (Figs 4 and. 5, [Fig f5]) confirm the bulk crosslinking. Although our electrocuring is similar to the free radical-based electropolymerization via reduction at the cathode^34^,^35^,^36^, the simultaneous crosslinking at both the surfaces of the cathode and anode has never been reported. That is, in the existing electropolymerization systems based on free radicals, the free radical induced crosslinking only occurs on the surface of working electrode and the polymerization reaction cannot propagate out into the bulk solution, and consequently, no adhesive network can be formed. While in our electrocuring system, two types of free radicals produced by electrochemical activation can act as multifunctional linkers for surface binding and the polymer crosslinking. This necessitates that the electrocuring be activated by sacrificial conducting electrodes (not necessarily metallic), but the surfaces themselves need not be conducting if interspersed or interdigitated electrodes are employed. Supplementary Figure 2 demonstrates these observations, where carbon electrodes, screen printed on a plastic tab (Zensor TE100 chips), were electrocured to non-conductive polyester terephthalate (PET) film (Supplementary Fig. 2a) and borosilicate glass coverslip (Supplementary Fig. 2b) substrates. Despite the large differences in surface properties between these substrates, adhesive strength was similar for both materials.

There are unique applications of electrocuring adhesives not possible with other on-demand adhesives. On-demand adhesion via electrocuring has the advantages of microcontroller activation, non-specific crosslinking and tunable material properties that open applications that are not currently possible with photo- or thermocuring adhesives. This would be particularly beneficial for conducting or non-conducting substrates that are opaque or thermally sensitive. Thermoset electrically conductive adhesives have become a key alternative method to replace solder as the bonding medium for components in printed circuit boards^37^. While this can replace lead-based solders, it still requires heating the substrates to high temperatures that preclude recent developments in bioelectronics or polymer electronics that involve soft-tissue interfaces^38^. Conductive electrocuring adhesives would be ideal in such situations. The exceptional ability of being able to choose the viscoelastic properties of the electrocuring adhesive may allow adhesive material properties to be refined for specific vibrational frequencies or match the mechanical properties of the substrates they are applied to, which is particularly important in the application of bioadhesives to dynamic substrates. Few technologies allow such predictable tuning of the storage or loss modulus (G′ and G″) or offer a method of electronic feedback for precise generation of G′/G″ ratios.

Little development in on-demand adhesives technology has limited the methods used for activation to thermocuring (thermosets), photocuring and chemical-curing adhesives. Here we demonstrate for the first time a method of curing an adhesive through low-voltage activation or ‘electrocuring'. The electrochemical mechanism of the electrocuring adhesive gives it unique properties—end users can tune both the material properties and adhesion strength at will—allowing tailored adhesives to be easily constructed for the application at hand. The electrocuring adhesive is synthesized with carbene precursor functional groups capped on a PAMAM dendrimer. Our results support the theory that low-voltage activation triggers the carbene groups to crosslink electrode surfaces and neighbouring dendrimers; however, other methods of crosslinking cannot be ruled out, such as azine-mediated crosslinking. Overall, our findings open a new class of electrocuring, on-demand adhesive that allows tunable material properties and new development on substrates that are not currently possible with photo- or thermocuring adhesives.

## Methods

### Synthesis of PAMAM-g-diazirine conjugate

Poly(amidoamine) dendrimer (fifth generation, 28.8 kDa) with 128 mol. eq. amino termini (PAMAM, Dendritech, Inc, USA) was diluted to 0.5% w/w PAMAM/methanol (137 μM PAMAM, 17.6 mM NH_2_). Aliquots of 3-[4-(bromomethyl)phenyl]-3-(trifluoromethyl)-diazirine (henceforth, aryl-diazirine) TCI, Tokyo) were directly added to 5 ml of 0.5% w/w PAMAM/methanol solution in the amounts of 15.5 mg (55.6 μmol), 31.0 mg (111 μmol), and 46.5 mg (167 μmol), for a theoretical yield of 5%, 10% and 15% mol/mol diazirine/PAMAM NH_2_ conjugation degree, respectively. Reaction was vigorously stirred for 36 h at room temperature and evaporated under vacuum to yield a pale yellow viscous liquid.

### Liquid chromatography tandem mass spectroscopy (LC–MS/MS)

Liquid chromatography and analysis were performed on a Shimadzu Series LC–MS/MS 8030 system equipped with a degasser (DGU-20A3), a binary pump (LC-20AD), an autosampler (SIL-20A HT) and a column oven (CTO-20AC). The detector of this system was a tandem quadrupole mass spectrometer combined with electrospray ionization. The analysis was carried on a C18 polar reversed-phase column (Synergi Fusion-RP, Phenomenex) with the particle size of 4 μm, the length of 150 mm and inner diameter of 2.0 mm. The column was guarded with a precolumn with 4 × 2.0 mm C18 security guard cartridge (Synergi Fusion-RP, Phenomenex). The column oven temperature was set to 25 °C. The mobile phase was methanol with 0.1% formic acid (B) and deionized water with 0.1% formic acid (A). The LC program was: holding 10% B for 2 min; then gradient increasing to 90% B in 38 min and holding for 10 min, immediately dropping to 10% B and holding for another 10 min to achieve column equilibrium for the next run. The flow rate of the mobile phase was 0.2 ml min^−1^. Samples (10-μl injection) were analysed by MS under full scan mode (*m*/*z* 50–500).

### SEC–MALLS–UV analysis of PAMAM-g-diazirine conjugates

A Wyatt MiniDawn three-angle light-scattering detector (Wyatt Technology Corperation, US), a 20A/20AV ultraviolet–visible detector (Shimadzu, Japan) and an Agilent 1100 refractive index detector (Agilent Technologies, Santa Clara, CA, USA) were incorporated in line with An Agilent 1100 series high-performance liquid chromatography pump complete with degasser and PLGel aqueous 50 (Agilent Technologies) in a 35 °C oven. PAMAM samples were dissolved in eluent buffer (1% w/v glacial acetic acid (166.7 mM) with 0.2% (w/v) NaN_3_ (30.8 mM) and injected (50 μl, 1 ml min^−1^ flow rate) at a concentration of 2 mg ml^−1^. PAMAM molar mass and diazirine conjugation degree were calculated using Wyatt ASTRA (version 5.19.1) software (Wyatt Technology, Santa Barbara, CA, USA) with the following parameters; PAMAM d*n*/d*c*=0.185 (ref. 39) and the ultraviolet extinction coefficient of 1,009.5 ml cm^−1^ g^−1^ at 350 nm, respectively.

### Electrochemistry of aryl-diazirine and PAMAM-g-diazirine

Electrochemical experiments were performed on an Autolab instrument (Metrohm, Netherlands) using a standard three-electrode system consisting of a Ag/AgCl electrode with saturated KCl aqueous internal filling solution (reference electrode), platinum wire (counter electrode), and glassy carbon (working electrode, diameter 3 mm for cyclic voltammetry, diameter 6 mm for chronopotentiometry). Aryl-diazirine (1.0 mM) was dissolved in acetonitrile with 0.1 M tetraethylammonium perchlorate ((C_2_H_5_)_4_NClO_4_) electrolyte. Acetic acid (0.1 wt%, 16.7 mM) was added as a proton donor in subsequent experiments and the electrostimulated product was analysed by LC–MS/MS. The PAMAM-g-diazirine was dissolved in phosphate buffer solution (PBS, pH 7.4, 0.1 M) at a concentration of 30.3 μM.

### Oscillatory rheometry of electrochemically activated PAMAM-g-diazirine

PAMAM-g-diazirine (100 μl dissolved in PBS at 25 wt%) was pipetted onto a disposable Zensor chip with three electrodes (Zensor R&D Company, Taipei, China). The loss modulus (G″) and the storage modulus (G′) of the PAMAM-g-diazirine hydrogel were analysed in a real-time parallel-plate rheometer set-up (Physica MCR 501 Rheometer, USA) under oscillatory dynamic analysis of 1% amplitude at 1 Hz with a 8-mm diameter stainless steel probe (PP08/150, Anton PAAR, USA) temperature controlled at 25 °C. The Probe was maintained at 100-μm gap away from the surface of the immobilized Zensor chip. The voltage between these electrodes was maintained with a portable potentiostat (pocketSTAT, Ivium Technologies, USA). The amplitude sweep of the PAMAM-g-diazirine hydrogels was analysed in real-time parallel-plate oscillatory rheometer set-up. The frequency for the amplitude swap was fixed as 1 Hz and the strain ranged from 0.01 to 30%.

### Shear adhesive strength measurements

The shear adhesive strength of PAMAM-g-diazirine was performed according to ASTM standard F2255–05 via ITO glass plates (Huananxiangcheng Technology Co., Ltd. Shenzhen, China). The ITO plates were firstly cut into 100 × 150 mm slides and ultrasonically cleaned for 15 min in each of the following solvents in order of acetone, ethanol and deionized water. One piece of the ITO plate was placed horizontally with two ends covered by two pieces of dielectric PTFE tape (20 × 110 mm, 90-μm thick) for thickness control. PAMAM-g-diazirine (20 μl dissolved in PBS at 25 wt%, 7.6 mM) was pipetted onto the ITO glass plate. This system was covered by another piece of ITO plate on top so that the PAMAM-g-diazirine solution distributes uniformly between these two ITO plates, which were powered by the portable potentiostat. PAMAM-g-diazirine was stimulated under −2 V and the shear stresses were recorded by TCD110 Series Force Measurement System (Chatillon Force Measurement Products, USA) at a controlled strain rate of 3 mm min^−1^.

PETfilm (80 μm) and glass coverslip (25.4 × 76.2 mm, 1–1.2-mm thick, CLP, China) were cut into 0.7 × 1.0 cm slides and then ultrasonically cleaned together with the Zensor electrode for 1 min in deionized water. PAMAM-g-diazirine (20 μl dissolved in PBS at 25 wt%, 7.6 mM) was pipetted in between the Zensor electrode and PET/glass slide with a 0.15 (0.06)-mm gap. This ‘sandwich structure' is demonstrated in Supplementary Fig. 2. The gel was stimulated under −2 V potential for 10 min via Zensor electrode powered by the portable potentiostat and the shear stresses were measured and recorded by TCD110 Series Force Measurement System with a controlled strain rate of 3 mm min^−1^.

## Additional information

**How to cite this article:** Ping, J. *et al.* Adhesive curing through low-voltage activation. *Nat. Commun.* 6:8050 doi: 10.1038/ncomms9050 (2015).

## Supplementary Material

Supplementary Information Supplementary Figures 1-4, Supplementary Table 1 and Supplementary References
