# Supplementary material for: Adhesive curing through low-voltage activation
Source: Nat Commun. 2015 Aug 18;6:8050. doi: 10.1038/ncomms9050 (PMC4557340; doi:10.1038/ncomms9050)
Supplement: Supplementary Information — Supplementary Figures 1-4, Supplementary Table 1 and Supplementary References [file ncomms9050-s1.pdf]

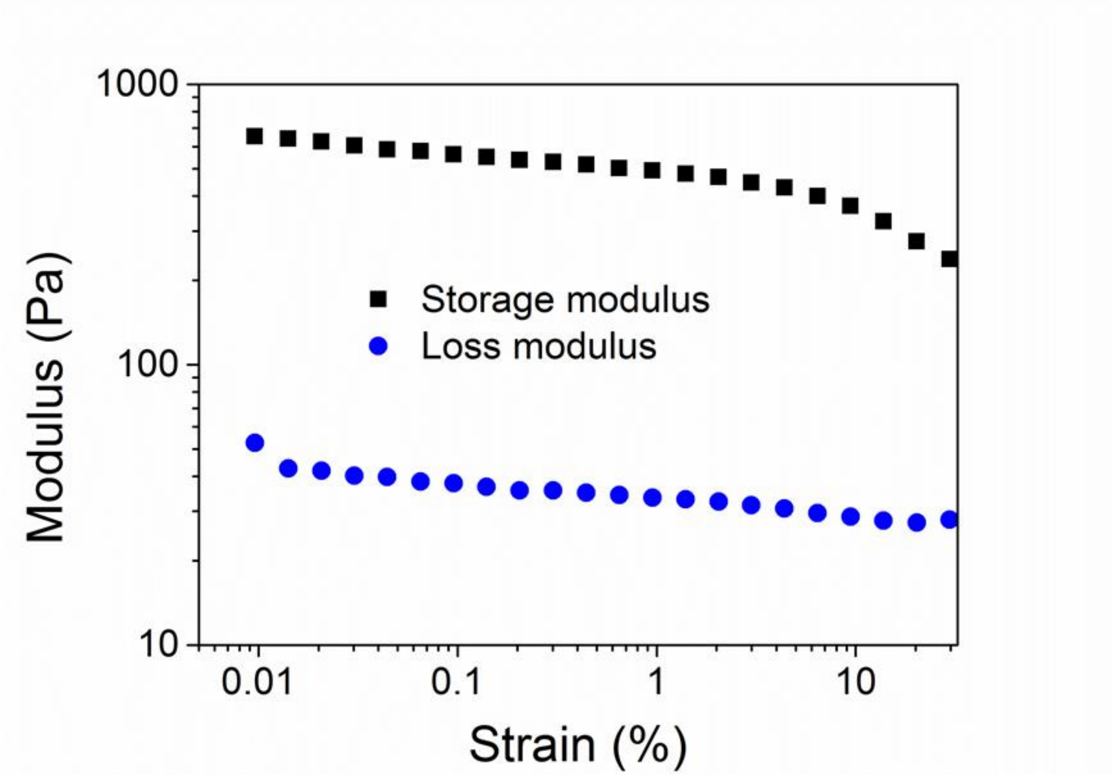

Supplementary Fig. 1 Amplitude sweep of electrochemically activated PAMAM-g-diazirine conjugate. Amplitude sweep test with a constant frequency as 1 Hz of PAMAM-g-diazirine conjugate (25 wt% in PBS in all figures) stimulated under -2.0 V vs. Ag/AgCl on the disposable Zensor chip for 5 min.

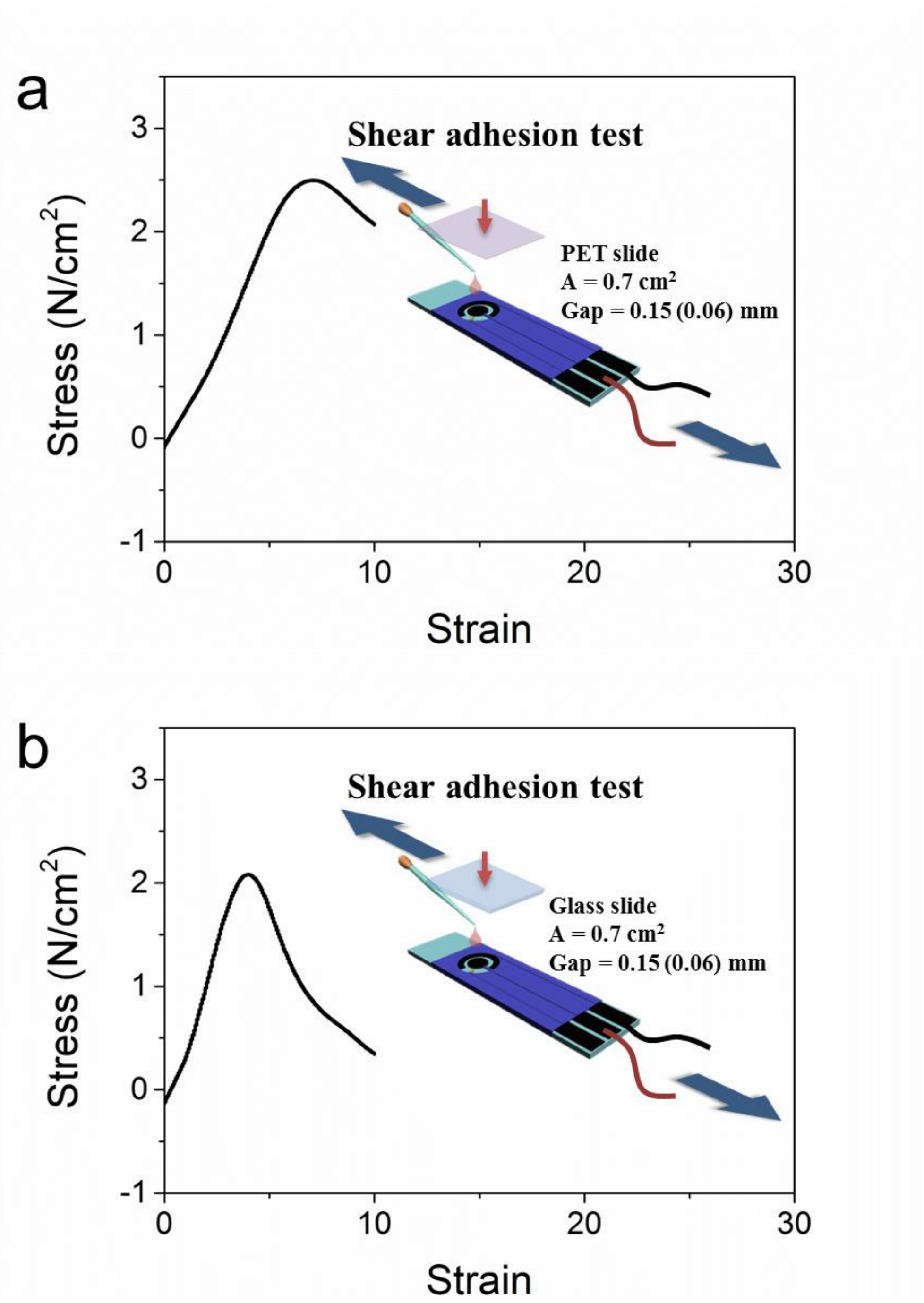

Supplementary Fig. 2 Shear adhesion ability of PAMAM-g-diazirine with non-metallic surface. (a) Stress and strain curve of PAMAM-g-diazirine (15% conjugation, 25 wt% in PBS) between Zensor electrode and PET film stimulated under -2.0 V potential for 10 min. (b) borosilicate glass coverslip.

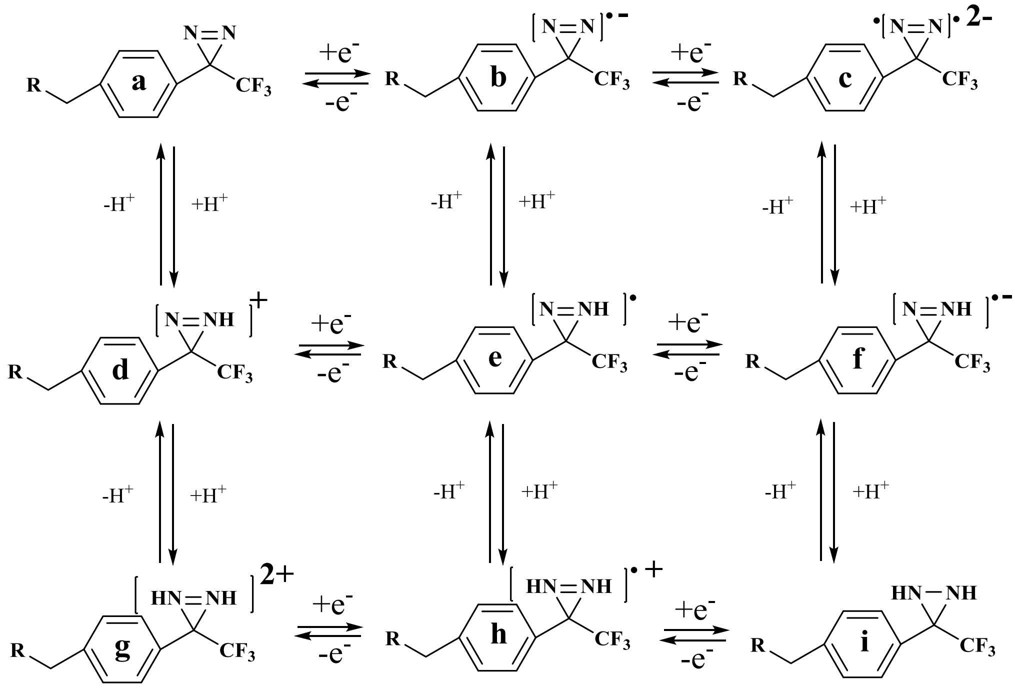

Supplementary Fig. 3 Square scheme of diazirine reduction to diaziridine. Path a → b is predominant as measured herein in Fig 1 and others[1,2]. By logical deduction, only paths befi or bcfi predominate, with preponderance of negatively charged species[3].

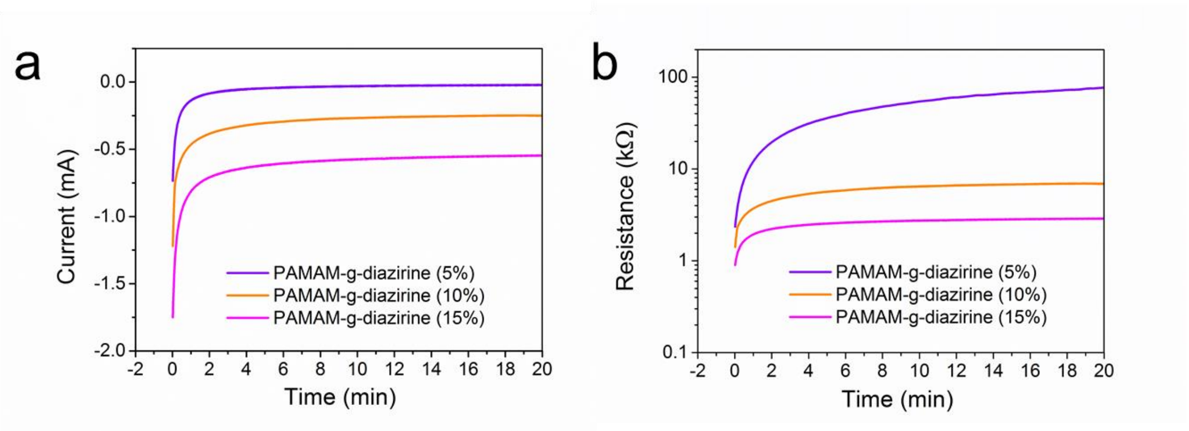

Supplementary Fig. 4 Current and resistance of PAMAM-g-diazirine under stimulation. (**a**) Current versus time from experiment in Fig. 3b. Current is shown to exponentially decrease due to the formation of the electrical double layer, electrografting of the PAMAM-g-diazirine, or combination thereof. (**b**) Resistance versus time, as calculated from the current by Ohm's Law.

Supplementary Table 1 Size exclusion chromatography multi-angle laser light scattering ultraviolet detector (SEC-MALLS-UV) analysis of PAMAM-g-diazirine.

Theoretical conjugation (%)[a]	Mass of PAMAM (μg)[b]	Mass of aryl-diazirine (μg)[c]	Peak Elution Volume (mL)[d]	Molar Mass (Da)[e]	Experimental conjugation (%)
0	165 ± 12	0.33 ± 0.42	8.63	28600 ± 400	0.16
5	206 ± 22	12.5 ± 0.6	8.56	31300 ± 500	4.8
10	189 ± 13	17.3 ± 0.7	8.52	33400 ± 500	9.3
15	134 ± 8	20.1 ± 0.6	8.38	35000 ± 600	14.4

[a] Theoretical and experimental conjugation refers to the percentage of amino groups on poly(amidoamine) modified with aryl-diazirine (PAMAM-g-diazirine).

[b] The mass of PAMAM was calculated according to dn/dc (0.185) and the refractive index signal.

[c] The conjugated mass of aryl-diazirine was calculated using the UV extinction coefficient at 350 nm after subtracting from PAMAM background signals.

[d] Molecules with larger molecular weight would have lower peak elution volume. Mean error is 0.01 mL or less.

[e] Weight averaged molecular weight calculated through laser light scattering and refractive index signal.

Supplementary References

1. Elson, C. M. & Liu, M. T. H. Electrochemical-Behavior of Diazirines. *J. Chem. Soc. Chem. Comm.* 415-416 (1982).
2. Elson, C. M., Liu, M. T. H. & Mailer, C. Electron-Spin-Resonance Studies of Diazirine Anion Radicals. *J. Chem. Soc. Chem. Comm.* 504-506 (1986).
3. Laviron, E. Electrochemical reactions with protonations at equilibrium: Part XII. The 1 e^-, 2 H^+ homogeneous isotopic electron exchange reaction (nine-member square scheme). *J. Electroanal. Chem.* **169**, 29-46 (1984).
